# The Impact of Nursing Education on Emergency Bedside External Ventricular Drain Insertion for Patients With Acute Hydrocephalus

**DOI:** 10.7759/cureus.34262

**Published:** 2023-01-27

**Authors:** Soha A Alomar, Sara T Bandah, Gaida A Noman, Mai Kadi, Ghada A Abulnaja, Ghadah Abdullah

**Affiliations:** 1 Department of Surgery, Division of Neurosurgery, King Abdulaziz University Faculty of Medicine, Jeddah, SAU; 2 Department of Community Medicine, King Abdulaziz University, Jeddah, SAU; 3 Department of Nursing Education, King Abdulaziz University Hospital, Jeddah, SAU; 4 Department of Nursing Administration, King Abdulaziz University Hospital, Jeddah, SAU

**Keywords:** care bundle, ventriculostomy, hydrocephalus, nursing, external ventricular drain, education

## Abstract

Objectives

Acute hydrocephalus is a neurosurgical emergency that requires immediate intervention. With emergency external ventricular drain (EVD) insertion and management, such rapid intervention can be a safe bedside procedure. Nurses play an integral role in patient management. Thus, this study aims to assess the knowledge, attitudes, and practices of nurses from different departments regarding bedside EVD insertion in patients with acute hydrocephalus.

Methods

EVD and intracranial pressure (ICP) monitoring competency checklists were developed, and a quasi-experimental, single-group, pre/post-test study was conducted at a university hospital in Jeddah, Saudi Arabia, in January 2018 during an educational program. The neurosurgery team determined program efficacy using pre/post-questionnaires. All attendees who agreed to fill in the pre- and post-survey and whose data were complete were included in the study.

Results

Of the 140 nurses who participated in the study, the data of 101 were analyzed. Knowledge level improved significantly between the pre- and post-test; for example, when asked about administering antibiotics before EVD insertion, the pre-test correct response rate of 65% increased to 94% in the post-test (p<0.001), and 98% considered the session informative. However, the attitude toward bedside EVD insertion did not change after the teaching sessions.

Conclusion

This study emphasizes the importance of ongoing nursing education, hands-on training, and strict adherence to an EVD insertion checklist to achieve successful bedside management of patients with acute hydrocephalus.

## Introduction

Acute hydrocephalus is a neurosurgical emergency that requires immediate intervention This condition can occur in both adult and pediatric patients with brain tumor, intracranial hemorrhage, head injury, and central nervous system infection. External ventricular drain (EVD) insertion is a lifesaving procedure for patients with acute hydrocephalus and one of the most frequently performed neurosurgical procedures [1]. An EVD is a catheter that is percutaneously introduced into the frontal horn of the lateral ventricle using a twist drill or burr hole and connected to an external transducer, which can be used for both diagnostic measuring of intracranial pressure (ICP) and therapeutic purposes such as cerebrospinal fluid (CSF) drainage [2].

Although EVD placement is associated with complications such as bleeding, obstruction, and infection, with infection rates reaching up to 22% [3], it can be a safe operating room (OR) or even bedside procedure as long as the involved personnel are well trained and have the required equipment. Multiple studies have demonstrated the efficacy of bedside EVD placement [4]. Lately, in most centers, EVD maintenance and surveillance of complications have become the nurses’ duty [5]. Thus, training the nursing staff and gaining their support for EVD placement may significantly increase the likelihood of success [6].

Regarding implementation, at Shands Hospital at the University of Florida, adherence to a ventriculostomy placement bundle including hand hygiene, antibiotics use, and strict sterile techniques resulted in a significant reduction in infection rates [7]. At our center, however, there are some limitations in bedside EVD insertion implementation mainly owing to nurses’ lack of knowledge about this procedure and the perception of high complication rates when performed outside the OR setting. In many situations, a quick deterioration in patient condition mandates bedside EVD insertion rather than waiting for OR availability, which could lead to irreversible complications.

This study aims to assess nurses’ general knowledge, awareness, and attitudes toward bedside EVD insertion in patients with acute hydrocephalus before and after participating in an education program. Perceived barriers to bedside EVD insertion are also assessed.

## Materials and methods

A quasi-experimental, single-group, pre/post-test study was conducted at a university hospital in Jeddah, Saudi Arabia, in January 2018. The King Abdulaziz University Ethics Committee provided ethical approval (reference number: 153-18). Oral informed consent was obtained from all participants.

Competency checklists for EVD insertion and ICP monitoring

In previous studies, evidence-based competency checklists for EVD insertion and ICP monitoring were developed to assist registered nurses and clinicians in providing safe and effective patient care and to offer guidance regarding patient assessment, preparation, intraoperative and postoperative care, and management [8-11]. The process in this study involved several stages. The first stage included meetings between the nursing education team, emergency room (ER) nursing team, and the neurosurgeon in charge to review the ICP monitoring policy and EVD guidelines and resources. The second stage involved cooperation between the education and ER nursing teams to create an EVD competency checklist based on the literature. The third stage was the development of a competency checklist for ICP monitoring, based on the literature and integrating hospital policy and guidelines. In the fourth stage, the checklists were reviewed, and feedback was given to the education and ER teams as the urgent needs for ICP monitoring and EVD may necessitate longer ER stays. In the final stage, the checklists were ready to use in the ER.

Education sessions and questionnaire distribution

The study population included nurses who attended the education program, consisting of six sessions one hour each, conducted over two days. The lectures included information about EVD, indications for EVD insertion, avoiding complications, patient assessment during the procedure and the aftercare, and familiarization with the nursing competency checklist. It also included a hands-on demonstration of the system setup and priming, as well as troubleshooting. Pre/post-questionnaires were distributed to all attendees. Information about the sessions was distributed to all nurses through the nursing managers and nursing education unit. All attendees in the six sessions were asked to fill in the forms pre- and post-sessions. Any participant who did not fill in the forms, fill one form only (pre or post), or did not answer all the questions was excluded from the analysis.

The questionnaire included 13 questions developed by the neurosurgeon and pilot tested on 10 participants who were not included in the main analysis. The first part covered demographic data and prior experience, the second included multiple-choice questions about knowledge and practical points related to EVD insertion and management, and the third assessed barriers to bedside EVD insertion and whether these education sessions improved nurses’ knowledge.

Data analysis

Statistical analysis was performed using Stata software release 14 (StataCorp LLC, College Station, Texas, USA). Categorical variables were summarized using counts and percentages and continuous variables using mean±standard deviation and median/interquartile range (IQR) for normally and non-normally distributed variables, respectively. The paired t-test and chi-square test were used to assess whether the average knowledge score and response distribution were significantly different between the two time points. P-values<0.05 were considered significant. We used McNemar’s test to assess the difference in the knowledge (high versus low) before and after the educational session.

## Results

Of the 140 nurses who were recruited, the data of 101 who completed the pre- and post-survey were included in the analysis (females: 86%; average age: 35.3±7.97 years). ER nurses represented 56.6% of the sample. The median period of practice was 10 (IQR: 5-15) years. Most nurses had never observed or assisted in EVD insertion (68.3%), while 19.8% had observed or assisted in ≤5 of such procedures, and only 4% had participated in >10 procedures (Table 1).

**Table 1:** Characteristics of nurses who participated in pre- and post-lecture survey ICU, intensive care unit; SD, standard deviation; EVD, external ventricular drain

Characteristic	N (%)
Gender	
Male	14 (14%)
Female	87 (86%)
Age mean (SD)	35.3 (7.9)
Years of practice mean (SD)	11.4 (7.8)
Department	
Emergency room	57 (56.60%)
Medical ICU	16 (16.10%)
Surgical ICU	5 (5.10%)
Pediatric ICU	4 (4%)
Pediatric floor	10 (10.10%)
Surgical floor	6 (6.10%)
Medical floor	2 (2%)
Number of EVD observed or assisted	
None	69 (68.30%)
1-5	20 (19.80%)
6-10	8 (7.90%)
>10	4 (4%)
The lecture was useful	
Yes	99 (98%)
No	2 (2%)

Table 2 presents the answers to knowledge questions pre- and post-lecture. When asked about the timing of antibiotic administration pre-lecture, 65% gave the correct response: within 30 minutes before the procedure. Post-lecture, 94% responded correctly (p<0.001). When asked what healthcare workers should wear during the procedure pre-lecture, 59.6% answered “hat and mask”; this percentage increased to 89% post-lecture (p<0.001). When asked what to do when changing the ventriculostomy draining bag pre-lecture, 14.3% indicated that despite the need for sterility, “there is no need to wear a mask and hat,” while only 7.4% said so post-lecture (p<0.002).

**Table 2:** Pre- and post-lecture responses to knowledge questions Responses for each question were summarized using counts and percentages, while the average knowledge score was summarized using mean±standard deviation (SD) §Statistical analysis was performed using McNemar’s test Bold: paired t-test correct answers EVD, external ventricular drain; HCP, healthcare provider

Knowledge question	Pre-lecture	Post-lecture	P-value§
Q6. Timing of antibiotics			<0.001
<1 hour	33 (33.0%)	6 (6.0%)	
<30 minutes	65 (65.0%)	94 (94.0%)	
Any time before or after depending on the situation	2 (2.0%)	0 (0.0%)	
Q7. During insertion			<0.001
Wear a hat and a mask	59 (59.6%)	89 (89.0%)	
Wear a mask	14 (14.1%)	2 (2.0%)	
A mask should be worn if the HCP will be near the sterile field	26 (26.3%)	9 (9.0%)	
Q8. Changing the bag			0.002
A hat, a mask, and sterile gloves	39 (39.8%)	62 (65.3%)	
A mask and sterile gloves	45 (45.9%)	26 (27.4%)	
The procedure should remain sterile, but you do not need to wear a mask and hat	14 (14.3%)	7 (7.37%)	
Q9. Access port scrubbing time			<0.001
For three minutes	35 (35.0%)	94 (94.9%)	
For 30 seconds to one minute	63 (63.0%)	5 (5.05%)	
However long the provider accessing the system scrubs	2 (2.0%)	0 (0.0%)	
Q10. During transfer			<0.001
Lowered below bed level	11 (11.0%)	1 (0.99%)	
Keep at the same level	12 (12.0%)	3 (2.97%)	
Clamped	74 (74.0%)	96 (95.0%)	
No change should be done	3 (3.0%)	1 (0.99%)	
Q11. Non-draining EVD			0.830
Flush the system	2 (2.0%)	1 (1.02%)	
Call neurosurgery	52 (52.0%)	54 (55.1%)	
Lower the drain to check for patency	46 (46.0%)	43 (43.9%)	
Answered both correct options	51 (51%)	52 (52%)	
Average knowledge score, mean (SD)	3.69±1.16	4.82±0.73	<0.001

On being asked about scrubbing the port pre-lecture, 35% said that it needed to be done for three minutes; post-lecture, 95% gave this answer (p<0.001). Regarding managing EVD during transfer pre-lecture, 74.3% said that it should be clamped; post-lecture, 95% gave this response (p<0.001).

Regarding EVD blockage management pre-lecture, 52% said that they would call a neurosurgeon, 46% said that they would lower the drain to check patency, and 51% chose both answers. These percentages did not differ significantly post-lecture at 55%, 44%, and 52%, respectively (p=0.83).

While the level of knowledge changed significantly between the pre- and post-test, attitudes and barriers perceived did not change. This is probably owing to a lack of relevant practical experience among most participants. However, 98% of the nurses reported that attending the lecture was helpful.

We classified the level of knowledge as high (4-6/6 questions correctly answered) and low (0-3/6 questions correctly answered). There was a significant difference in knowledge levels: 14.85% had poor knowledge before the program compared to 0.99% after it. Further, 28.71% had a high level of knowledge before the program, which subsequently rose to 78.21% (p<0.0001).

**Figure 1:**
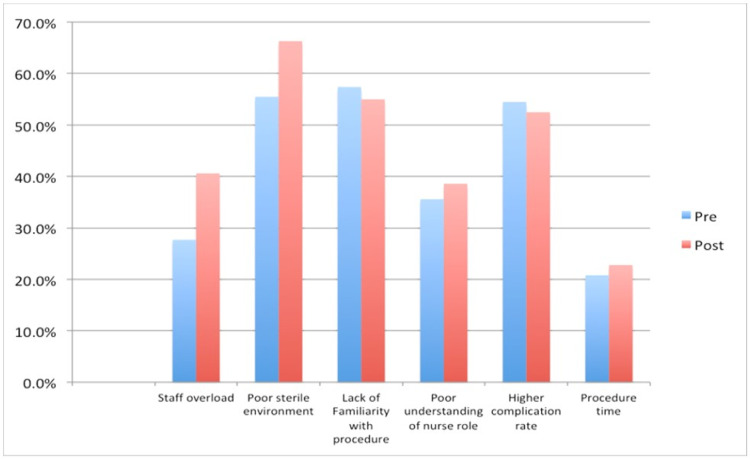
Perceived nurses’ barriers against bedside insertion of EVD pre- and post-lecture EVD: external ventricular drain

Age, years of experience, department, and number of EVD procedures observed were not associated with the mean knowledge score (p-values: 0.48, 0.92, 0.36, and 0.53, respectively). When asked about barriers to bedside EVD insertion, most nurses mentioned staff overload, poor sterile environment, and higher complication rates (Figure 1). Poor sterile environment was the most commonly perceived barrier. The perception that staff overload is the main barrier in bedside procedures became significantly stronger post-lecture (p=0.05). The post-test showed that fewer participants thought of the following reasons as barriers: familiarity of the procedure and complication rate (Figure 1).

## Discussion

External ventricular drain insertion is common in critically ill patients with sudden neurologic deterioration. The most common indication is acute hydrocephalus resulting from conditions such as subarachnoid hemorrhage, intraventricular hemorrhage, tumors obstructing the CSF pathway, infected shunts, and meningitis or to measure and control ICP in traumatic head injury. Owing to the urgency of this procedure, sometimes, there is a lack of OR availability, which, together with the patient’s instability, mandates bedside insertion. Bedsides, insertion is common in neurocritical care units due to the relative simplicity of the procedure [12].

Nurses are the first-line agents for both recognizing and managing life-threatening events as they handle most patient care responsibilities [13]. In the intensive care unit (ICU), it is the nurses’ responsibility to monitor and document ICP readings of patients, which requires proper skill and training [14]. Efficient and safe bedside EVD placement can be achieved by the collaboration between nurses and physicians to establish clear policies and protocols. Hands-on training for nurses involved in EVD care is essential in minimizing complications. Lwin et al. implemented protocols for EVD insertion and management for clinicians and nurses, which resulted in a significant reduction in EVD infection rate from 6.1% to 3.8%. Thus, the rate of EVD-related infections could be lowered by implementing protocols and bundles to improve nurses’ knowledge of and compliance with the standards of care [6,15]. What most of these protocols have in common is a strict sterile technique during insertion and handling, minimizing the frequency of accessing the system for flushing or sample collection, and prophylactic antibiotic use prior to EVD insertion [9].

There are concerns that bedside EVD insertion involves higher complication rates, particularly infections, which can prolong hospital stay, worsen neurologic status, increase costs, and lead to poor patient outcomes [16]. However, strict compliance with EVD bundles and protocols could significantly lower infection rates [17]. Nurses’ adherence to these protocols has been known to lead to lower EVD-related complications in many centers [18].

The American Association of Neuroscience Nurses’ guidelines have been proven effective in preventing EVD-related infections [6]. Many centers have developed bundles and protocols proven to lower the risk of infection similar to what we have seen in procedures such as central lines [19]. At Riverside Methodist Hospital in Central Ohio, there was no reported EVD infection for almost two years after EVD bundle application [20].

Studies show that complication rate is not affected by the location of EVD insertion. For example, Clark et al. retrospectively studied 175 cases of ICP monitor insertion in 140 patients with traumatic head injury and found minor differences in the incidence of infection between OR and ICU insertion (18.8% versus 8.4%, p=0.05) [21]. Bekar et al. reported the same finding in 631 patients, with no difference in infection rate in OR versus ICU insertion (5.34% versus 4.28%, p=0.05) [22]. Regarding hemorrhage rates, Gardner et al. found no difference in intracranial hemorrhage in 188 patients with EVD regardless of OR or ICU insertion (34.8% versus 44.3%, p=0.21) [23]. Ehtisham et al. also showed that there is no difference with regard to infection rate between EVD and ICP monitors placed in the OR versus bedside in the neurocritical care unit as all cases remained infection-free [24]. Thus, the location of EVD placement does not seem to influence complication rates.

In general, prior studies have not addressed nurses’ knowledge of and attitudes toward bedside EVD insertion. While a few studies have considered nursing awareness and knowledge about caring for traumatic head injury including ICP monitoring, they have not focused specifically on EVD care. We, therefore, created a checklist outlining nursing responsibilities before, during, and after EVD insertion. Then, we conducted on-site lectures aiming to increase nurses’ awareness of and familiarity with EVD insertion and aftercare. To determine the impact, we conducted a pre- and post-program survey that covered nurses’ knowledge, attitudes, and practice regarding bedside EVD insertion.

The bundle contained a checklist of the essential steps for proper EVD insertion and ICP monitoring. Our survey showed that 68.3% of the participants had never observed or assisted in EVD insertion by an expert (Table 1), while 19.8% had observed or assisted in ≤5 EVD insertion procedures, and only 4% had participated in >10 procedures. This limited practical experience in managing patients and assisting physicians during EVD insertion is considered a major barrier to the implementation of bedside EVD insertion.

The level of knowledge improved significantly in the post-lecture survey. However, attitudes and barriers did not change significantly, indicating some resistance from the nurses toward the implementation of bedside EVD placement. This could be due to concerns about work overload and potentially increased infection rates, as well as the previously mentioned lack of practical experience in observing or assessing during the procedure. Hence, we believe that the development of EVD bundles and protocol and continuous intense hands-on training experience could improve nurses’ attitudes toward bedside EVD insertion.

When asked about barriers to bedside EVD insertion, most participants listed patient overload, staff shortage, poor sterile environment, and higher complication rates. To address this issue, we could apply these bundles and perform EVD insertion outside the OR only in certain allocated areas in the hospital such as adult and pediatric ICUs, where there is a 1:1 patient-to-nurse ratio and a higher level of nursing skills in dealing with invasive monitoring.

In his investigation of critical care nurses’ management of patients with head injury, one of the aspects Kiewiet studied was nurses’ knowledge about ICP monitoring [25]. The results indicated that there is no correlation between knowledge about head injury care and demographic characteristics, such as gender, age, nursing and ICU care qualification, clinical discipline, overall years of experience, and critical care experience [25]. Even though managing and interpreting ICP monitors are only some of the aspects of caring for patients with severe traumatic head injury, the author reached the same conclusion as many other studies that two aspects are essential in improving patient care and outcomes: improving knowledge and practical skills for nurses through frequent lectures and seminars and developing guidelines and protocols to minimize confusion among healthcare providers and avoid inconsistency [25].

There are different methods used to provide educational material for nursing staff. Mehmood et al. implemented an “EVD care bundle” through face-to-face meetings and posters; consequently, EVD infection rates were significantly reduced, from 27% to 10% (p<0.001) [26]. In a study that used a coconut to simulate an infant skull, “mock herniations” were conducted to evaluate nurses’ skills in setting up an emergent EVD. Both time and accuracy improved remarkably, proving that periodic practice can improve response times and accuracy [18,27].

In our study, we found no relation between participants’ age, years of practice, department, and number of EVD procedures observed (p-values: 0.48, 0.92, 0.36, and 0.53, respectively) and knowledge scores.

Limitation

This study is limited by the small sample size; further studies with more nurses from multiple departments should be conducted. Further, as the setting was a single institution, the findings might not be widely generalizable.

Future directions

We aim to more comprehensively assess the impact of educational sessions on EVD insertion outside the OR on nurses with more hands-on experience. Longitudinal prospective studies are needed to compare complication rates between bedside and OR insertion of EVD.

## Conclusions

We emphasize the importance of education and hands-on training for nurses, facilitated by creating an EVD checklist for the bedside management of acute hydrocephalus by EVD insertion. However, improvements in knowledge levels do not necessarily correspond with changes in attitudes or practice. Thus, there is a need for intensive continuous education with hands-on experience. The implementation of bundles with the consistency of practice is essential for lowering these barriers and improving nurses’ attitudes toward bedside EVD insertion.

## Appendices

Table 3 shows the checklist of the essential steps for proper EVD insertion and ICP monitoring.

**Table 3:** External ventricular drain checklist: nursing responsibilities before, during, and after the insertion EVD, external ventricular drain; GCS, Glasgow Coma Scale; BP, blood pressure; CBC, complete blood count; INR, international normalized ratio; PTT, partial thromboplastin time; PT, prothrombin time; CSF, cerebrospinal fluid; MD, medical department; Q, every; ER, emergency room

External ventricular drain checklist	Achieved	Not achieved	Comment
Nursing responsibilities before the insertion of EVD: patient assessment			
Perform hand hygiene before patient contact			
Introduce yourself to the patient			
Verify the correct patient using two identifiers			
Perform a baseline neurologic assessment to include the level of consciousness (GCS score of 8), mental status, motor and sensation cranial nerves, neurologic injury (such as systemic trauma), abnormal brain CT, and vital signs (systolic BP <90 mmHg)			
Obtain the patient’s medical and surgical histories, including obstructive hydrocephalus, subarachnoid hemorrhage, cerebral edema, meningitis, and surgical mass lesion, and obtain medication history (anticoagulant and anti-platelet)			
Review the patient’s current laboratory profile to assess coagulation status, including CBC, platelet count, PT, PTT, and INR			
Assess for allergies			
Patient preparation			
Ensure that the patient and family understand the procedural teaching. Ensure that informed consent has been obtained. Verify the correct patient with two identifiers			
Initiate intravenous access, or assess the potency of the intravenous line			
Perform hand hygiene, and don personal protective equipment (PPE): sterile gown, gloves, surgical cap, and mask			
Prime the system with preservative-free normal saline before attaching to the patient’s catheter to prevent the collection of air bubbles that will change the flow of fluid to the collection bag, which cause an inaccurate reading. Ensure that priming the system should be done with a sterile technique. Ensure that all tubing connections remained sterile when connecting and flushing			
Nursing responsibilities during the insertion of EVD: assisting with the insertion of an EVD			
Perform hand hygiene before patient contact			
Apply PPE (gloves, gowns, and mask with eye shields). After opening the outer packaging, apply sterile gloves to handle sterile supplies			
Assist the patient to a supine position with the head of the bed at 30 degrees and the patient’s head and neck in a neutral position. Align the torso and the lower extremities			
Administer sedation and analgesia as prescribed			
Assist as needed with antiseptic preparation of the insertion site			
Connect the monitoring system to the distal tip of the catheter after it is inserted			
Assist as needed with the application of the catheter stabilization sterile and sterile occlusive dressing			
Nursing responsibilities post-insertion of EVD			
Label and arrange for sending CSF sample if collected			
Label the EVD tubing (to avoid confusion with peripheral or central lines)			
Ensure all valves are opened and the system is draining			
Place the EVD at the level requested by the MD in relation to ear level (i.e., 0, 5, 10, 15, or 20 cm above the external auditory canal)			
Clamp EVD during patient transfer and turning or bed elevation to avoid over drainage			
Ensure EVD is opened once patient transfer is completed			
Monitor EVD output Q hourly while the patient is in the ER (is it feasible? If not, Q four hourly is the maximum)			
Check for CSF leak around the dressing Q four hourly			
Inform MD if the output is zero/hour or more than 20 cc/hour			
